# An NMR-Based Metabolomic Approach to Investigate the Effects of Supplementation with Glutamic Acid in Piglets Challenged with Deoxynivalenol

**DOI:** 10.1371/journal.pone.0113687

**Published:** 2014-12-11

**Authors:** Miaomiao Wu, Hao Xiao, Wenkai Ren, Jie Yin, Jiayu Hu, Jielin Duan, Gang Liu, Bie Tan, Xia Xiong, Abimbola Oladele Oso, Olayiwola Adeola, Kang Yao, Yulong Yin, Tiejun Li

**Affiliations:** 1 Scientific Observing and Experimental Station of Animal Nutrition and Feed Science in South-Central China, Ministry of Agriculture, Hunan Provincial Engineering Research Center of Healthy Livestock Key Laboratory of Agro-ecological Processes in Subtropical Region, Institute of Subtropical Agriculture, Chinese Academy of Sciences, Changsha, Hunan, China; 2 University of Chinese Academy of Sciences, Beijing, China; 3 Department of Animal Nutrition, College of Animal Science and Livestock Production, Abeokuta, Ogun State, Nigeria; 4 Department of Animal Sciences, Purdue University, West Lafayette, United States of America; National Research Council of Italy, Italy

## Abstract

Deoxynivalenol (DON) has various toxicological effects in humans and pigs that result from the ingestion of contaminated cereal products. This study was conducted to investigate the protective effects of dietary supplementation with glutamic acid on piglets challenged with DON. A total of 20 piglets weaned at 28 d of age were randomly assigned to receive 1 of 4 treatments (5 piglets/treatment): 1) basal diet, negative control (NC); 2) basal diet +4 mg/kg DON (DON); 3) basal diet +2% (g/g) glutamic acid (GLU); 4) basal diet +4 mg/kg DON +2% glutamic acid (DG). A 7-d adaptation period was followed by 30 days of treatment. A metabolite analysis using nuclear magnetic resonance spectroscopy (^1^H-NMR)-based metabolomic technology and the determination of superoxide dismutase (SOD) and glutathione peroxidase (GSH-Px) activities for plasma, as well as the activity of Caspase-3 and the proliferation of epithelial cells were conducted. The results showed that contents of low-density lipoprotein, alanine, arginine, acetate, glycoprotein, trimethylamine-N-oxide (TMAO), glycine, lactate, and urea, as well as the glutamate/creatinine ratio were higher but high-density lipoprotein, proline, citrate, choline, unsaturated lipids and fumarate were lower in piglets of DON treatment than that of NC treatment (*P<*0.05). Compared with DON treatment, dietary supplementation with glutamic acid increased the plasma concentrations of proline, citrate, creatinine, unsaturated lipids, and fumarate, and decreased the concentrations of alanine, glycoprotein, TMAO, glycine, and lactate, as well as the glutamate/creatinine ratio (*P<*0.05). Addition glutamic acid to DON treatment increased the plasma activities of SOD and GSH-Px and the proliferating cell nuclear antigen (PCNA) labeling indexes for the jejunum and ileum (P<0.05). These novel findings indicate that glutamic acid has the potential to repair the injuries associated with oxidative stress as well as the disturbances of energy and amino acid metabolism induced by DON.

## Introduction

The trichothecene mycotoxin deoxynivalenol (DON) is often found as a contaminant in agricultural staples, and the toxic effects of DON have been well-characterized in humans as well as pigs which is the most susceptible animal [1]. Due to the high percentage of grain in the pig diet, pigs can easily be exposed to DON. If we consider the similarities between humans and pigs, the pig can be regarded as a good model for investigating the toxicity of DON in humans [2]. Although the pathogenesis caused by DON in vivo is being revealed, little is known about the metabolic mechanism of DON in piglets.

While many strategies have been developed to reduce the toxic effects of DON, including physical adsorption, chemical decomposition and microbial detoxification [3]–[5], dietary strategies are the most promising approach to the problem[6]. Glutamic acid, a functional amino acid, is one of the most abundant amino acids in intestinal tract protein [7]. This nutrient plays multiple roles in the intestine, including energy production [8], taste activation [9], metabolism[10], redox state and detoxification process [11]. Thus,glutamic acid may be useful for alleviating the injury to the intestine induced by DON. Indeed, we have found that supplementation with glutamic acid can alleviate the negative effects of DON in piglets. However, little is known about the mechanism by which glutamic acid exerts its beneficial effects in DON-challenged piglets.

Substantial effort is being directed toward the study of metabolomics, which provides a useful systematic approach to understanding the global metabolic responses of living systems to influence such as disease, nutrition and environment [12]–[14]. Thus, the plasma metabolome that is associated with treatment with glutamic acid and DON in piglets was determined by a nuclear magnetic resonance-based (NMR) metabolomic method. This study was conducted to analyze the toxic effects of DON-contaminated feed on pigs and to investigate the effects of supplemental glutamic acid on DON-induced toxic damage in piglets.

## Materials and Methods

This study was conducted in accordance with the Chinese Guidelines for Animal Welfare and was approved by the Animal Care and Use Committee of the Chinese Academy of Sciences (Beijing, China) [15].

### Preparation of DON-contaminated feed

The mold strain *F.graminearum* R6576, that is only able to produce DON, was provided by the College of Plant Science & Technology of Huazhong Agricultural University, China. DON-contaminated feed was prepared according to previous reports from our group [6]. The resulting feed was determined to contain 4mg/kg DON [16], and the dose was chosen according to the report by Prelusky et al. (1994). Processed diet was mixed with unprocessed diet at a ratio of 1∶1, and glutamic acid was added according to the experimental design.

### Experimental design

A total of 20 piglets (Duroc × Landrace × Large Yorkshire) weaned at 28 d of age were randomly assigned to receive 1 of 4 treatments (5 piglets/treatment): 1) basal diet, negative control (NC); 2) basal diet +4 mg/kg DON (DON); 3) basal diet +2% (g/g) glutamic acid (GLU); 4) basal diet +4 mg/kg DON +2% (g/g) glutamic acid (DG). The basal diets were prepared from corn, soybean meal, wheat bran, limestone, CaHPO_4_, salt, and additive premix to meet or exceed the nutritional requirements for growing pigs as recommended by the NRC (1998) (Table 1). The amount of DON (mg/kg) in the NC, DON, GLU, and DG diets was determined to be 1.02±0.03, 4.01±0.06, 1.03±0.02, and 4.03±0.04, respectively.

**Table 1:** Composition and nutrient levels of basal diet (as-fed basis)^1^.

Item	NC	DON	GLU	DG
Ingredients, %				
Corn	61.25	61.25	60.03	60.03
Soybean	15.79	15.79	15.47	15.47
Extruded-soybean	10.00	10.00	9.80	9.80
Fish meal	5.00	5.00	4.90	4.90
Wheat bran	3.00	3.00	2.94	2.94
Soybean oil	1.74	1.74	1.71	1.71
Premix^2^	1.00	1.00	0.98	0.98
Limestone powder	0.98	0.98	0.96	0.96
Calcium hydrogen phosphate	0.78	0.78	0.76	0.76
Salt	0.37	0.37	0.36	0.36
Glutamic acid	0.00	0.00	2.00	2.00
Lys·HCl (98%)	0.09	0.09	0.09	0.09
Analyzed chemical composition				
DM, %	89.85	89.84	89.83	89.82
CP, %	18.90	18.91	18.96	18.97
Crude ash, %	6.79	6.78	6.77	6.75
Calculated DE, kcal/kg	3400	3400	3400	3400

1 NC  =  uncontaminated basal diet, DON  =  basal diet contaminated with deoxynivalenol (4 mg/kg), GLU  =  uncontaminated basal diet supplemented with 2% glutamic acid; DG  =  DON diet supplemented with 2% glutamic acid.

2 Providing the following amount of vitamins and minerals per kilogram on an as-fed basis: Zn (ZnO), 50 mg; Cu (CuSO_4_), 20 mg; Mn (MnO), 55 mg; Fe (FeSO_4_), 100 mg; I (KI), 1 mg; Co (CoSO_4_), 2 mg; Se (Na_2_SeO_3_), 0.3 mg; vitamin A, 8,255 IU; vitamin D_3_, 2,000 IU; vitamin E, 40 IU; vitamin B_1_, 2 mg; vitamin B_2_, 4 mg; pantothenic acid, 15 mg; vitamin B_6_, 10 mg; vitamin B_12_, 0.05 mg; vitamin PP, 30 mg; folic acid, 2 mg; vitamin K_3_, 1.5 mg; biotin, 0.2 mg; choline chloride, 800 mg; and vitamin C, 100 mg. The premix did not contain additional copper, zinc, antibiotics, or probiotics.

The experiment was arranged as a randomized design, and pigs were allowed free access to water throughout the experimental period. After an adaptation period of 7 days, piglets were fed their respective diets 3 times per day (at 8:00, 13:00 and 18:00) for a 30-d period. Fifteen and 30 d after the initiation of treatment, 10 mL of blood was collected from a jugular vein into a collection tube with heparin sodium 2 h after feeding, and centrifuged at 1000×g for 10 min at 4°C to obtain plasma samples, which were stored at −80°C for further analysis. On d 30, piglets were anesthetized with sodium pentobarbital and exsanguinated. The small intestine was excised, and rinsed thoroughly with ice-cold physiological saline solution, and the jejunum and ileum were dissected out. Two-centimeter segments of the mid-jejunum and mid-ileum were cut and fixed in 4% formaldehyde for measurements of crypt cell proliferation. In addition, samples of the jejunal and ileal mucosa were immediately snap-frozen in liquid N and stored at −80°C for the determination of Caspase-3.

### Plasma GSH-Px and SOD activities

Glutathione peroxidase (GSH-Px) and superoxide dismutase (SOD) activities were measured using spectrophotometric kits in accordance with the manufacturer's instructions (Nanjing Jiancheng Biotechnology Institute, Nanjing, China).

### Intestinal Crypt Cell Proliferation and caspase-3

After tisssue samples were subjected to dehydration, embedding, and sectioning, crypt cell proliferation was determined using proliferating cell nuclear antigen (PCNA) as described by Xu et al. (2003). [17] The primary monoclonal antibody against PCNA (Calbiochem, Cambridge, UK) was obtained commercially (Wuhan Boster Biological Technology Co., Ltd., Wuhan, China) together with a streptavidin-biotin complex detection kit. Prior to staining, the sections were heated by microwave in 0.01 M citric acid solution for antigen retrieval. As a negative control, primary antibodies were replaced with phosphate buffer solution. The stained sections were reviewed and scored independently by 2 investigators using a microscope (Olympus, Tokyo, Japan). The PCNA labeling index was expressed as the ratio of cells that were positively stained for PCNA to all epithelial cells in at least 5 areas that were randomly selected for counting at less than 200-fold magnification. Caspase-3 activity was measured using a colorimetric assay kit in accordance with the manufacturer's instructions (Keygentec, Nanjing, China).

### ^1^H NMR Spectroscopic measurement of plasma samples

^1^H NMR spectroscopic measurement of plasma samples was conducted as described previously [18]. Briefly, plasma samples (500 µL) were placed in 5-mm NMR tubes with 50 µL D2O (as a lock signal) and 50 µL 0.9% saline. All NMR spectra were measured at a ^1^H frequency of 600.11 MHz using a Bruker Avance AVIII 600 spectrometer at 298 K (Bruker Biospin, Rheinstetten, Germany). Water pre-saturation was achieved by using a standard one-dimensional (1D) NMR spectrum, which is a general representation of the total metabolite composition. An inter-pulse delay of 3 µs, a mixing time of 100ms and irradiation of the water resonance were used to attenuate signals from macromolecules by the CPMG (Carr-Purcell-Meiboom-Gill) pulse sequence. Large macromolecule signals were detected by using a BPP-LED (bipolar-pair longitudinal eddy) current pulse sequence. For resonance assignment purposes, two-dimensional ^1^H-^1^H COSY (correlation spectroscopy) and TOCSY (total correlation spectroscopy) were also performed for selected plasma samples.

Free induction decays (FID) were multiplied by an exponential window function of 1.0 Hz prior to Fourier transformation and corrected for phase and baseline distortions using TopSpin 2.0 (Bruker). Chemical shifts were referenced to the peak of the anomeric proton of α-glucose at δ 5.23. NMR spectra (δ 0.5–8.5) were binned with regions 0.002 ppm wide and automatically integrated with the AMIX package (v.3.8.3, Bruker Biospin, Germany). The region at δ 4.55–5.13 was removed to avoid the effects of imperfect water suppression. Consequently, the spectra over the ranges δ 0.5–4.55, and δ 5.13–8.5 were selected and reduced to 3663 regions, each of which was 0.002ppm wide. Each integral region was normalized to the sum of all integral regions for each spectrum prior to pattern recognition analysis.

An overview of the data distribution and intersample similarities (e.g., clusterings and outliers) for each serum sample was first investigated by PCA (principal component analysis) using Simca-P 11.0 software (Umetrics, Sweden). NMR spectral data were further analyzed using the OPLS-DA (orthogonal projection to latent structure with discriminant analysis) method with unit variance scaling. Since the OPLS-DA results for the BPP-LED spectra of serum are similar to those for standard 1D spectra, the analysis of BPP-LED spectra will not be discussed in the Results section.

### Statistical Analysis

All statistical analyses were performed using the SAS software package (Version 9.2; SAS Institute, Cary, NC, USA). Data were subjected to a Proc Mixed analysis of variance-covariance followed by Tukey's multiple comparisons test. Data are expressed as the mean ± standard error of the mean.

## Results

### Plasma SOD and GSH-Px activities

**Figure 1:**
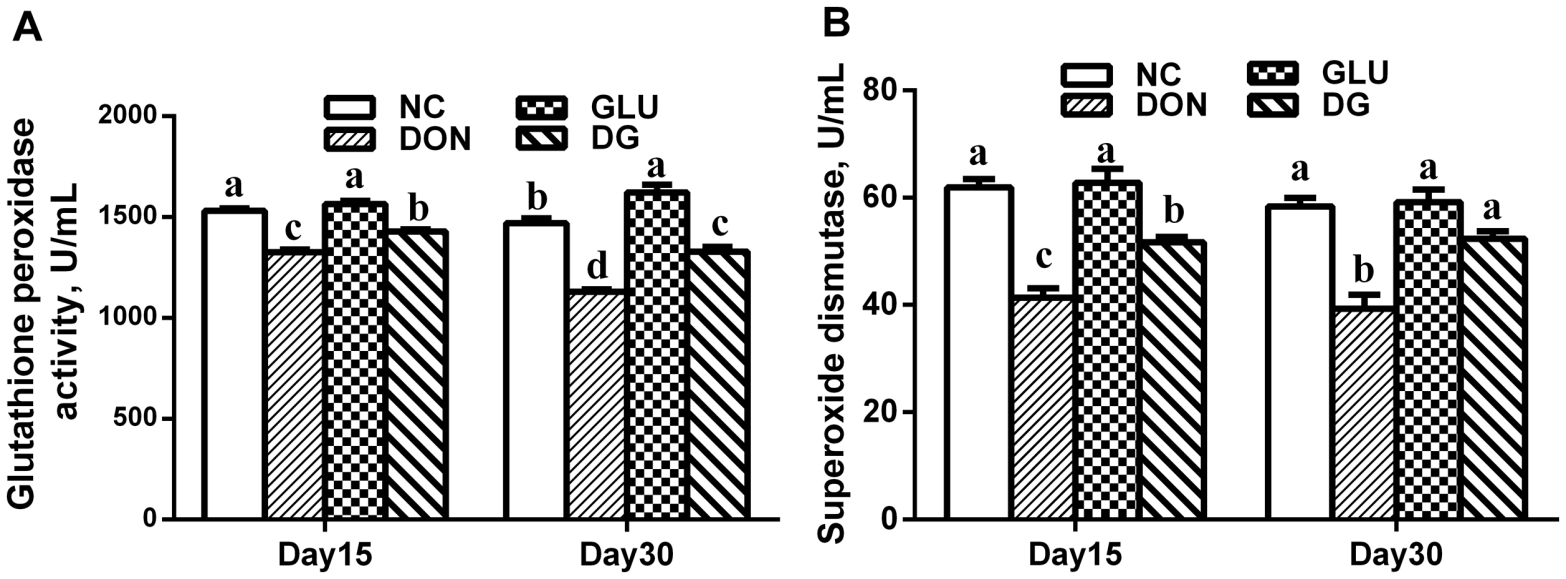
Antioxidant enzymes activities in each group. A: SOD activity in each group at day 15 and 30. B: GSH-Px activity in each group at day 15 and 30. Dietary treatments were NC, an uncontaminated basal diet, DON, the basal contaminated with 4mg/kg deoxynivalenol, GLU, uncontaminated basal diet with 2% glutamic acid supplementation, and DG, deoxynivalenol-contaminated (4 mg/kg) basal diet with 2% glutamic acid supplementation. Data are presented as means ± SEM, n = 5 for treatments, with a-d used to indicate statistically significant difference (*P<0.05*, one way ANOVA method). SOD: superoxide dismutase (U/ml); GSH-PX: glutathione peroxidase (U/ml).

Oxidative stress has been shown to be involved in the progression of DON-induced injuries, and investigations have found that dietary supplementation with DON increases the production of reactive oxygen metabolities, such as hydroxyl radical, hydrogen peroxide, and superoxide [19]. Furthermore, glutamic acid plays a crucial role in the intestinal tract as a regulator of oxidative reactions [11]. Thus, we determined the activities of two major factors in the anti-oxidative system: SOD and GSH-Px. As shown in Fig. 1, there was no significant difference in SOD between the NC and GLU groups on days 15 and 30 (Fig. 1 A), or in GSH-Px on day 15 (Fig. 1 B). However, treatment with GLU increased (*P<*0.05) GSH-Px activity on day 30, compared to that in the NC group (Fig. 1 B). DON decreased (*P<*0.05) SOD and GSH-Px activities compared with those in the control on days 15 and 30. This decrease was reversed (*P<*0.05) by supplementation with glutamic acid on days 15 and 30 (Figs. 1 A and B).

### Intestinal crypt cell proliferation and caspase-3

A decrease in the production of PCNA labeling index has been reported in piglets treated with DON [20]. In this study, we found that the DON significantly reduced (P*<*0.05) the PCNA labeling index in the jejunum and ileum, compared to that in the NC group (Table 2). The PCNA labeling index in both the jejunum and ileum in the DG group was greater than that with DON (*P<*0.05). Caspases orchestrate the dismantling and clearance of apoptotic cells, and among these caspase-3 appears to be responsible for the apoptotic hallmarks [21]. A significant increase in caspase-3 activity was observed in the jejunum and ileum of piglets in the DON group (*P<*0.05; Table 3). The caspase-3 activity in the jejunum and ileum in the GLU and DG groups did not differ from that in the NC group.

**Table 2:** Effects of glutamic acid on the proliferating cell nuclear antigen labeling index in the jejunum and ileum in piglets challenged with deoxynivalenol (%)^1^^,^ 2.

Item	Diet				SEM	*P-*value
	NC	DON	GLU	DG		
Jejunum	39.83^a^	6.15^c^	37.83^a^	29.48^b^	3.48	*<*0.01
Ileum	61.88^a^	22.63^c^	57.58^a^	50.13^b^	4.01	*<*0.01

a-c Values with different letters within the same row are different (*P*<0.05, one way ANOVA method).

1 Dietary treatments were NC, an uncontaminated basal diet, DON, the basal contaminated with 4mg/kg deoxynivalenol, GLU, uncontaminated basal diet with 2% glutamic acid supplementation, and DG, deoxynivalenol-contaminated (4 mg/kg) basal diet with 2% glutamic acid supplementation (n = 5).

2 Proliferating cell nuclear antigen labeling was defined as the ratio of positive cells to total cells in each section.

**Table 3:** Effects of glutamic acid on the Caspase-3 activity in the jejunum and ileum in piglets challenged with deoxynivalenol (%)^1^.

Item	Diet				SEM	*P-*value
	NC	DON	GLU	DG		
Jejunum	5.74^b^	7.60^a^	5.53^b^	5.50^b^	0.24	*<*0.01
Ileum	5.42^b^	7.33^a^	5.63^b^	5.75^b^	0.22	*<*0.01

a-b Values with different letters within the same row are different (*P*<0.05, one way ANOVA method).

1 Dietary treatments were NC, an uncontaminated basal diet, DON, the basal contaminated with 4mg/kg deoxynivalenol, GLU, uncontaminated basal diet with 2% glutamic acid supplementation, and DG, deoxynivalenol-contaminated (4 mg/kg) basal diet with 2% glutamic acid supplementation (n = 5).

### ^1^H NMR Spectroscopic measurement of plasma samples

**Figure 2:**
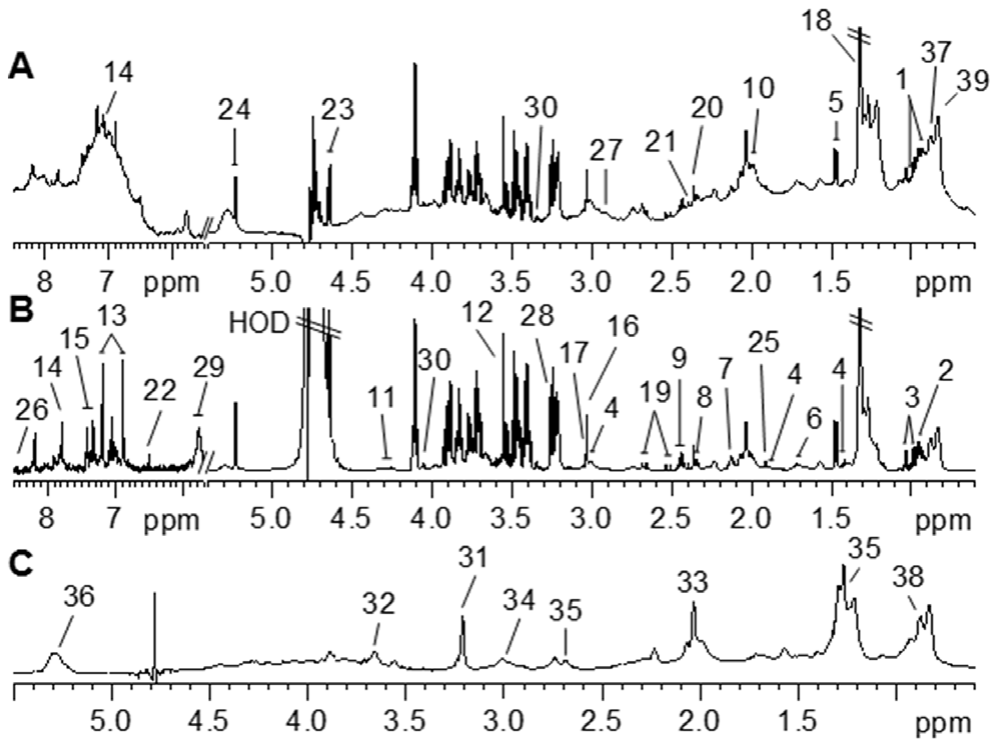
Typical 600 MHz ^1^H NMR spectra of plasma taken from piglets from standard 1D (A), CMPG (B) and BPP-LED (C) experiments. The spectra in the aromatic region were magnified four times (A) (δ 5.7–8.5) or eight times (B) (δ 5.7–8.5) compared to the aliphatic region (δ 0.6–5.4). Keys for metabolites are given in *Table 4*.

^1^H NMR spectra of biological fluids and tissues provide a unique fingerprint of the metabolic state of an organism along with considerable information on the nature of the drug or toxin to which an animal has been exposed [22], [23]. Examples of a ^1^H NMR CPMG (Fig. 2 A), standard 1D (Fig. 2 B), and BPP-LED (Fig. 2 C) spectra from a representative control pig fed the uncontaminated basal diet are shown in Fig. 2. In these spectra, 39 metabolites were unambiguously assigned by comparison to the published literature[12], [18], [24]–[26]. These assignments were confirmed by two-dimensional ^1^H-^1^H COSY and TOCSY methods (data not shown).

Visual inspection of the ^1^H NMR spectra revealed visible differences in plasma metabolites among piglets in the NC, DON, GLU and DG groups. For example, the concentrations of LDL, alanine, arginine, glycoprotein, TMAO, lactate and urea were higher, while those of HDL, proline, citrate, unsaturated lipids and fumarate were lower in the plasma from the DON group compared with that from the NC group. To perform a more detailed analysis of metabolic differences among the piglets in the four groups, multivariate data analyses were performed using PCA and OPLS-DA.

**Figure 3:**
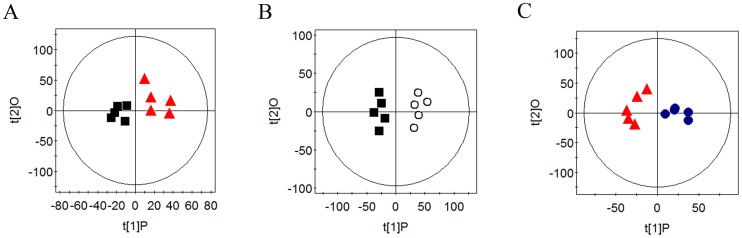
OPLA-DA scores for CPMG spectra of NC (▪), DON (▴), DG (•) and GLU (○) groups. Dietary treatments were NC, an uncontaminated basal diet, DON, the basal contaminated with 4 mg/kg deoxynivalenol, GLU, uncontaminated basal diet with 2% glutamic acid supplementation, and DG, deoxynivalenol-contaminated (4 mg/kg) basal diet with 2% glutamic acid supplementation.

PCA of plasma CMPG and standard 1D spectral data of piglets in the four groups showed clear clustering (data not shown). Further analysis using OPLS-DA indicated that the concentrations of plasma HDL, proline, acetate, citrate, unsaturated lipids, and fumarate were decreased (*P<*0.05) in the DON group, compared with those in the NC and GLU groups, while the concentrations of LDL, alanine, arginine, glycoprotein, TMAO, glycine, lactate, and urea, as well as the glutamate/creatinine ratio were increased (*P<*0.05) in the DON group, compared with those in the NC and GLU groups. Concentrations of plasma proline, citrate, unsaturated lipids, and fumarate were higher (*P<*0.05) in the DG group than in the DON group, and concentrations of plasma alanine, arginine, TMAO, glycine, and lactate, as well as the glutamate/creatinine ratio were lower (*P<*0.05) in the DG group (Fig. 3 and Table 4).

**Table 4:** Relative integrals (%)^1^ from selected plasma metabolites in piglets of all groups.

Metabolites		Relative integrals (%)^1^				SEM	*P*-value
		NC	DON	GLU	DG		
HDL(δ0.83)	CPMG	1.971^b^	1.751^c^	2.270^a^	2.007^ab^	0.091	<0.01
	Standard 1D	1.926^a^	1.814^b^	1.948^a^	1.848^b^	0.014	<0.01
LDL (δ0.84)	CPMG	1.010^b^	1.266^a^	0.964^b^	1.261^a^	0.036	<0.01
	Standard 1D	1.001^b^	1.062^a^	0.992^b^	1.051^a^	0.008	<0.01
Alanine (δ1.48)	CPMG	1.033^b^	1.237^a^	0.980^b^	1.016^b^	0.026	<0.01
	Standard 1D	1.013^b^	1.040^a^	1.003^bc^	0.993^c^	0.005	<0.01
Arginine (δ1.63)	CPMG	0.502^b^	0.658^a^	0.499^b^	0.610^ab^	0.022	<0.01
	Standard 1D	1.249^b^	1.314^a^	1.248^b^	1.304^a^	0.009	<0.01
Acetate (δ1.92)	CPMG	0.172^b^	0.218^a^	0.194^b^	0.173^b^	0.005	<0.01
	Standard 1D	0.310^b^	0.318^a^	0.320^ab^	0.318^ab^	0.005	0.168
Proline(δ2.00)	CPMG	1.204^a^	0.987^b^	1.185^a^	1.083^ab^	0.024	<0.01
	Standard 1D	1.478^a^	1.408^c^	1.486^a^	1.450^b^	0.008	<0.01
Glycoprotein (δ2.05)	CPMG	2.190^b^	2.766^a^	2.254^b^	2.630^a^	0.063	<0.01
	Standard 1D	1.461^c^	1.593^a^	1.460^c^	1.513^b^	0.014	<0.01
Citrate (δ2.52)	CPMG	0.196^a^	0.166^b^	0.187^a^	0.197^a^	0.004	<0.01
	Standard 1D	0.463^a^	0.451^b^	0.464^a^	4.681^a^	0.002	<0.01
TMAO (δ3.26)	CPMG	0.575^b^	0.811^a^	0.549^b^	0.613^b^	0.029	<0.01
	Standard 1D	0.169^b^	0.227^a^	0.184^b^	0.183^b^	0.006	<0.01
Glycine (δ3.56)	CPMG	1.179^b^	1.439^a^	1.237^b^	1.242^b^	0.028	<0.01
	Standard 1D	0.343^b^	0.380^a^	0.347^b^	0.337^b^	0.005	<0.01
Lactate (δ4.11)	CPMG	2.462^bc^	3.817^a^	2.316^c^	3.154^ab^	0.158	<0.01
	Standard 1D	1.289^b^	1.549^a^	1.234^b^	1.260^b^	0.034	<0.01
Unsaturated lipids (δ5.31)	CPMG	0.706^a^	0.576^b^	0.712^a^	0.678^ab^	0.018	0.014
	Standard 1D	0.757^a^	0.640^c^	0.781^a^	0.679^b^	0.014	<0.01
Urea (δ5.78)	CPMG	0.178^b^	0.271^a^	0.178^b^	0.244^a^	0.010	<0.01
	Standard 1D	0.165^c^	0.212^a^	0.179^b^	0.204^a^	0.005	<0.01
Fumarate (δ6.52)	CPMG	0.0061^a^	0.0033^b^	0.0061^a^	0.0057^a^	0.00003	<0.01
	Standard 1D	0.028^a^	0.026^b^	0.028^a^	0.028^a^	0.000	0.016
Glutamate/ Creatinine	CPMG	3.300^b^	4.152^a^	3.352^b^	3.435^b^	0.086	<0.01
	Standard 1D	3.265	3.253	3.252	3.248	0.023	0.995

1 Data are presented as means ± SEM, n  =  5 for treatments, with a-c used to indicate statistically significant difference (*P<0.05*, one way ANOVA method). Normalized integral of metabolites in spectrum (normalized to 100, chemical shift region over the ranges of δ 0.5–4.55 and δ 5.13–8.5.

Dietary treatments were NC, an uncontaminated basal diet, DON, the basal contaminated with 4 mg/kg deoxynivalenol, GLU, uncontaminated basal diet with 2% glutamic acid supplementation, and DG, deoxynivalenol-contaminated (4 mg/kg) basal diet with 2% glutamic acid supplementation (n = 5).

## Discussion

Among various mycotoxins, including DON, aflatoxin B1, zearalenone, fumonisin, fusariotoxin T2, and ochratoxin A, DON is encountered at the highest concentrations in the cereal foods worldwide [27], [28]. The toxic effects of DON in piglets have been studied in animal and cell culture experiments [20], [29], [30]. Previous studies have indicated that both short-term and subchronic exposure to DON disrupts immune function, antioxidative capacity, macromolecular synthesis, cell signaling, proliferation, gene up-regulation, and programmed cell death [19], [20], [30], [31]. Oxidative stress and reactive oxygen species may contribute to DON-induced toxicity in cells [32]–[34]. The present results regarding the plasma activities of anti-oxidative enzymes indicate that supplementing of DON-contaminated piglet diet with glutamic acid could significantly decrease the accumulation of reactive oxygen species (ROS) in the small intestine caused by DON. A possible explanation for this result is that glutamic acid is a precursor for glutathione, which is involved in the enterocyte redox state and in detoxification in enterocytes [35]. Thus, dietary supplementation with glutamic acid helps to scavenge the excess ROS induced by DON, thereby improving the balance between the production of ROS and the biological defense against the toxicity of these oxidants.

Due to its emetic effects, DON has been associated with human gastroenteritis [1]. It has been demonstrated that both chronic ingestion and dietary supplementation with DON induce morphological changes in the intestine of piglets, especially in the ileum and jejunum, as evidenced by shorter villi and a reduced number of goblet cells [20], [36]. The present changes in the intestinal morphology with DON and DG were supported by the PCNA labeling index, which was reduced in the DON group but increased in the DG group. PCNA is a suitable marker of proliferation potential, and is essential for DNA replication and repair [37], [38]. Mitochondria are very sensitive to oxidative stress damage, and it has been demonstrated that an excess of ROS can induce mitochondrial dysfunction [39]. It has been demonstrated that DON can induce caspase-3 activation and apoptosis in many cells [40], [41]. Similar to previous reports, the results of the current study indicate that glutamic acid can alleviate apoptosis in the small intestine induced by DON. A possible explanation is that glutamate is not only a precursor for enterocyte citrulline synthesis, but is also an ATP-producing substrate for enterocytes [35], [42]. In addition, glutamic acid is a precursor of *N*-acetylglutamate in enterocyte mitochondria, which is known to be an activator of the first step in citrulline biosynthesis. The plasma citrulline concentration has been proposed to be a reliable parameter for estimating the functional capacity of the intestine [43]–[45], and has been shown to correlate with enteral tolerance and bowel length in infants with short-bowel syndrome [46]. As a result, glutamic acid can be used within the intestinal mucosa to protect the intestinal epithelial barrier.

To further our understanding of the biological phenomena observed in the four treatment groups, we decided to perform targeted metabolomic analyses on a series of metabolites from the main metabolic pathways. These metabolites are the end-products or intermediates of cellular processes and therefore reflect the global integrated response of an organ or entire biological system to pathophysiologic stimuli [22]. Plasma metabolomic analysis revealed that many metabolites changed after exposure to DON. For example, treatment with DON resulted in a significant (P*<*0.05) increase in LDL and glycoprotein, and a marked decrease in HDL and unsaturated lipids, which suggested the presence of the lipid metabolism disorders. The results of the present study demonstrate for the first time that dietary supplementation with glutamic acid relieves the changes in the concentrations of lipids in the plasma of piglets, which is associated with the treatment of cardiovascular disorders. Triglycerides and cholesterol are transported by VLDL from the liver to various tissues. The triglycerides in VLDLs are hydrolyzed by lipase generating LDL. HDLs are involved in reverse cholesterol transfer from the tissues back to the liver. Total LDL and HDL particle concentrations have been used to assess the risk of cardiovascular disease [47], [48] and type 2 diabetes [49]. Our results indicate that LDL and HDL concentrations in the DON group were strongly and positively associated with atherogenic factors, while those with glutamic acid were inversely associated with such factors.

Another intriguing observation from the current study is that the DON group had an elevated concentration of TMAO and a greater glutamate/creatinine ratio, which suggested renal medullary injury and hepatic failure [50]. Several studies have demonstrated that TMAO is a marker of oxidative stress [51], is correlated with the degree of renal injury inflicted by different mechanisms [52], and is related to functions of the gut microbiota [53]. For example, TMAO in urine was associated with more intense medullary damage, and was also detected in clinical situations as acute toxic exposure due to xenobiotics, or under experimental exposure to nephrotoxins, where the increased excretion of TMAO is associated with medullary damage [54]–[56]. While dietary glutamic acid reduces the plasma TMAO concentration, a possible explanation is that glutamic acid has a significant beneficial effect on intestinal barrier function, and 95% of the dietary glutamic acid that is metabolized in the first pass contributes greatly to intestinal energy generation. Notably, TMAO is a microbial metabolite of carbohydrates and amino acids, which are likely produced in the lumen of the intestine [57]. Changes in this metabolite may result from an altered activity and/or reduced number of intestinal microorganisms [58]. This finding raises a crucial question regarding the role of glutamic acid in regulating nutrient metabolism and the ecology of the gut microbiota. In addition, plasma metabolomic analysis revealed that many other metabolites changed after exposure to DON, and dietary glutamic acid counteracts these changes. For example, treatment with DON resulted in an increase in lactate alanine. Lactate is an intermediate product of the citric acid cycle, and the abnormal metabolism of lactate is a hallmark of energy metabolic disorders. Metabolic disturbance of alanine is related to the dysfunction of glomerular filtration and recycling. An elevated concentration of acetate in the plasma suggests the presence of energy metabolism disorders related to an increase in ketone bodies. Dietary glutamic acid in enterocytes is a precursor for several other amino acids, including alanine, proline, and aspartate. Dietary glutamate is an important nutritional source of C and N, which enter the citric acid cycle and are metabolized mainly into CO_2_, lactate and amino acids [35].

Taken together, our findings demonstrated that DON induces oxidative stress, causes epithelial cell apoptosis, and induces energy, lipid and amino acid metabolism disorders. Furthermore, dietary supplementation with glutamic acid decreases oxidative stress, promotes intestinal epithelial cell proliferation, and regulates the metabolism disorders induced by DON, indicating that glutamic acid may be a useful nutritional supplement for regulating DON-induced injury.
